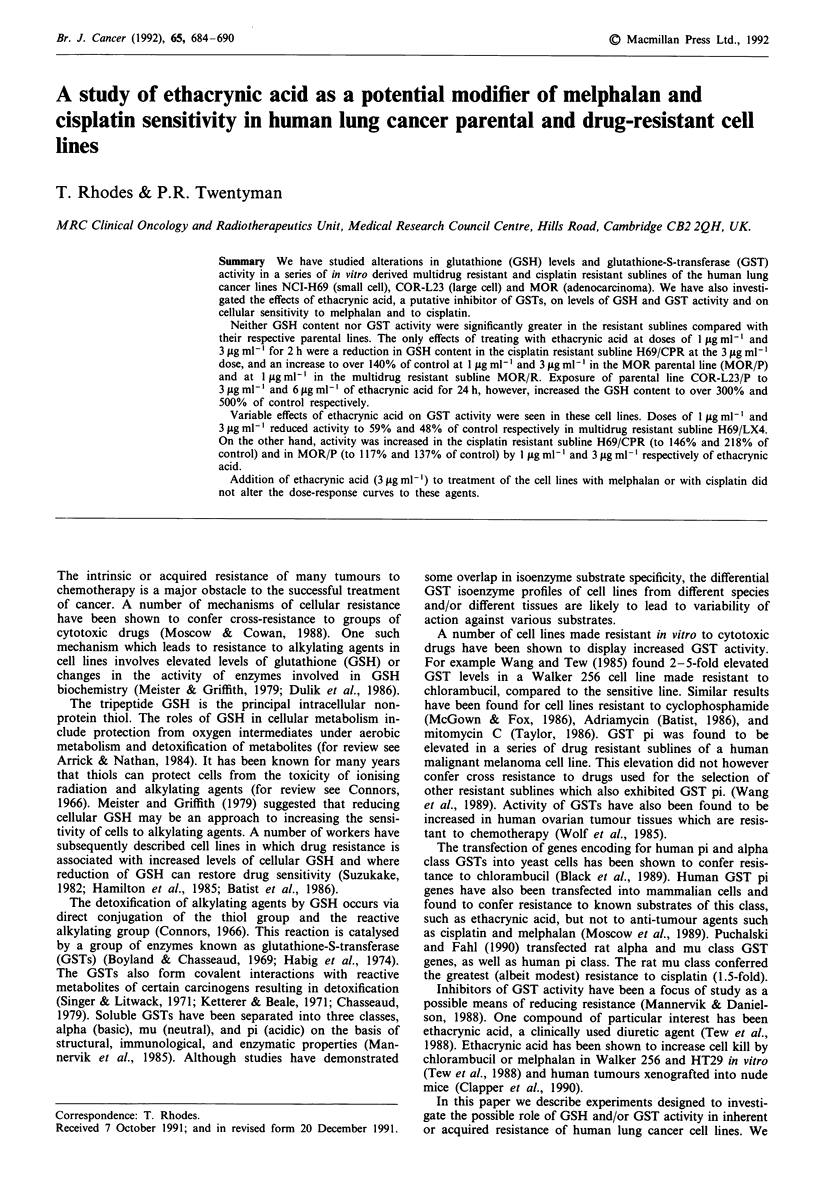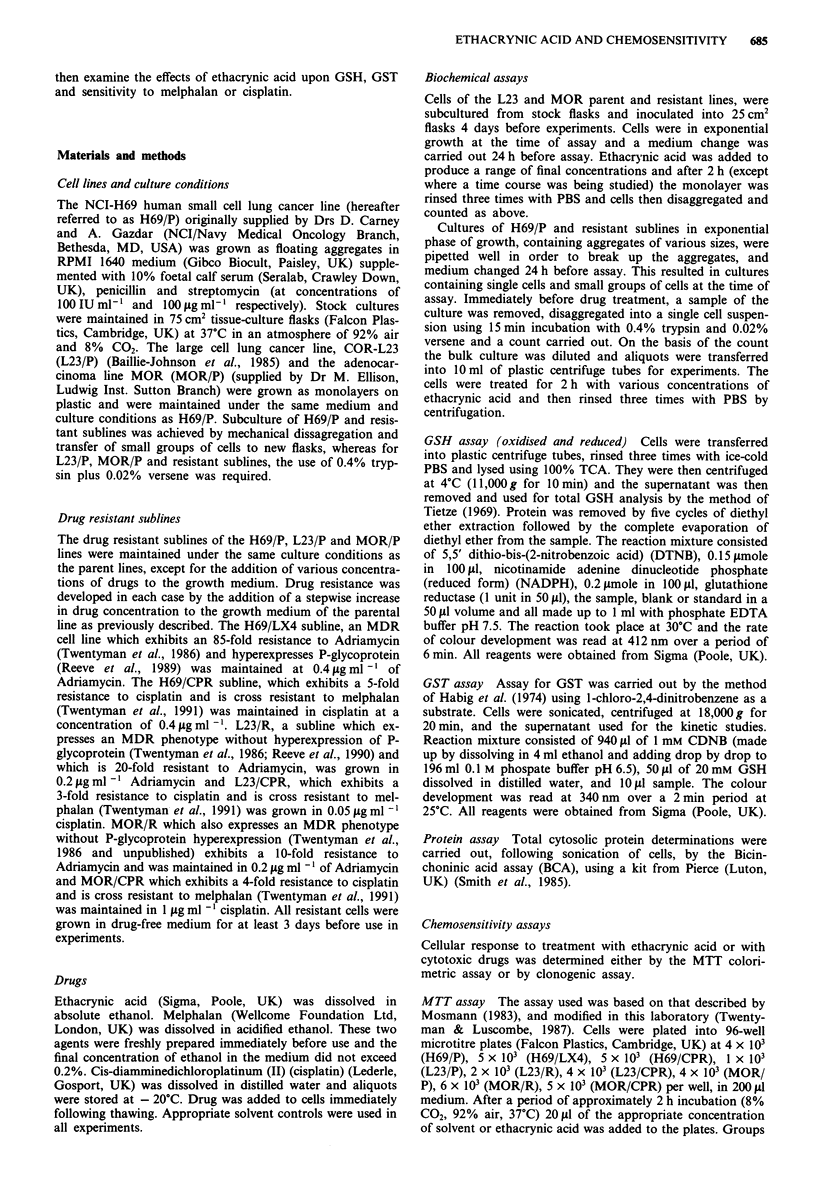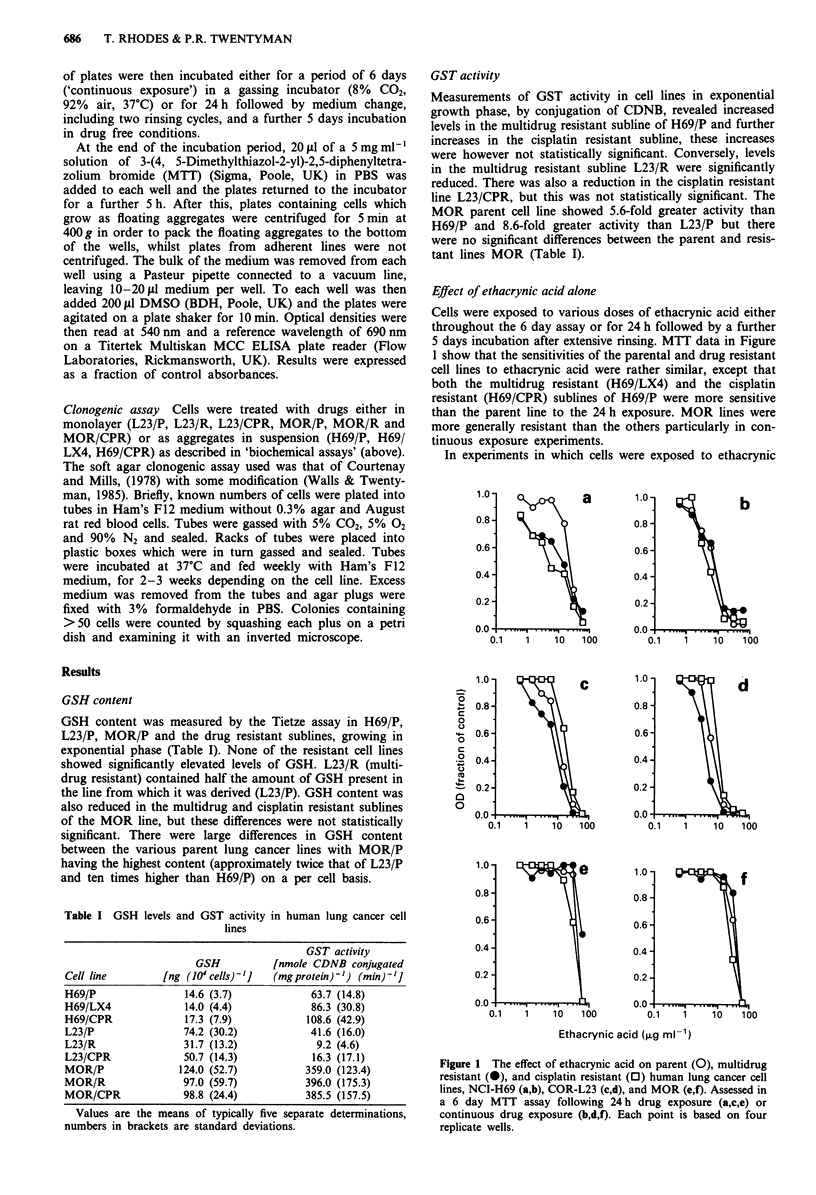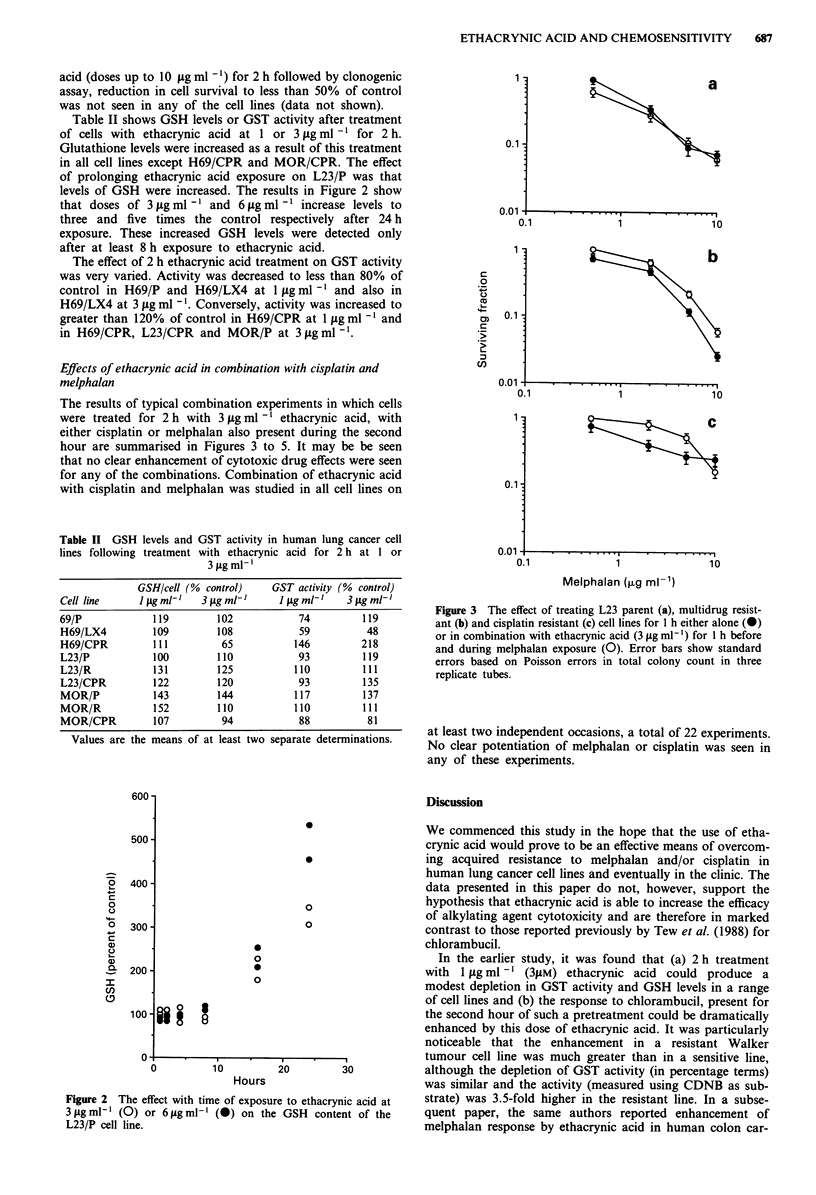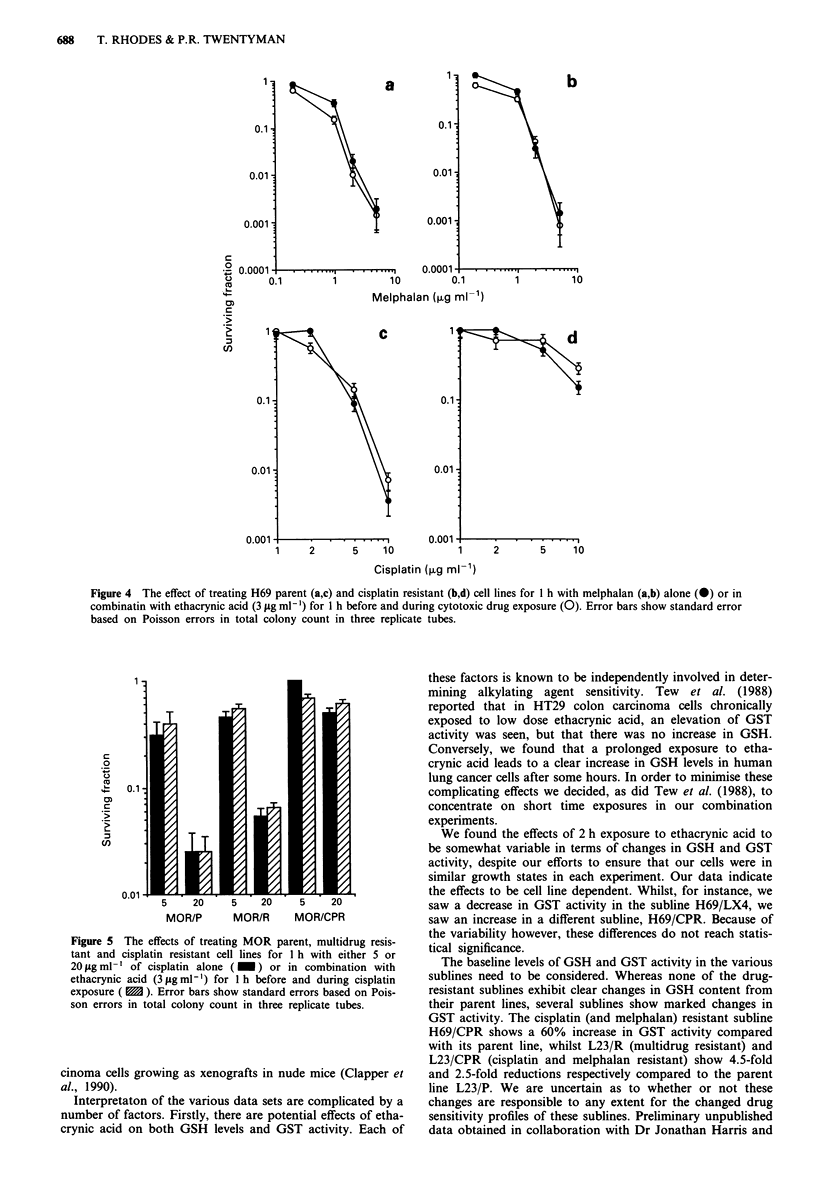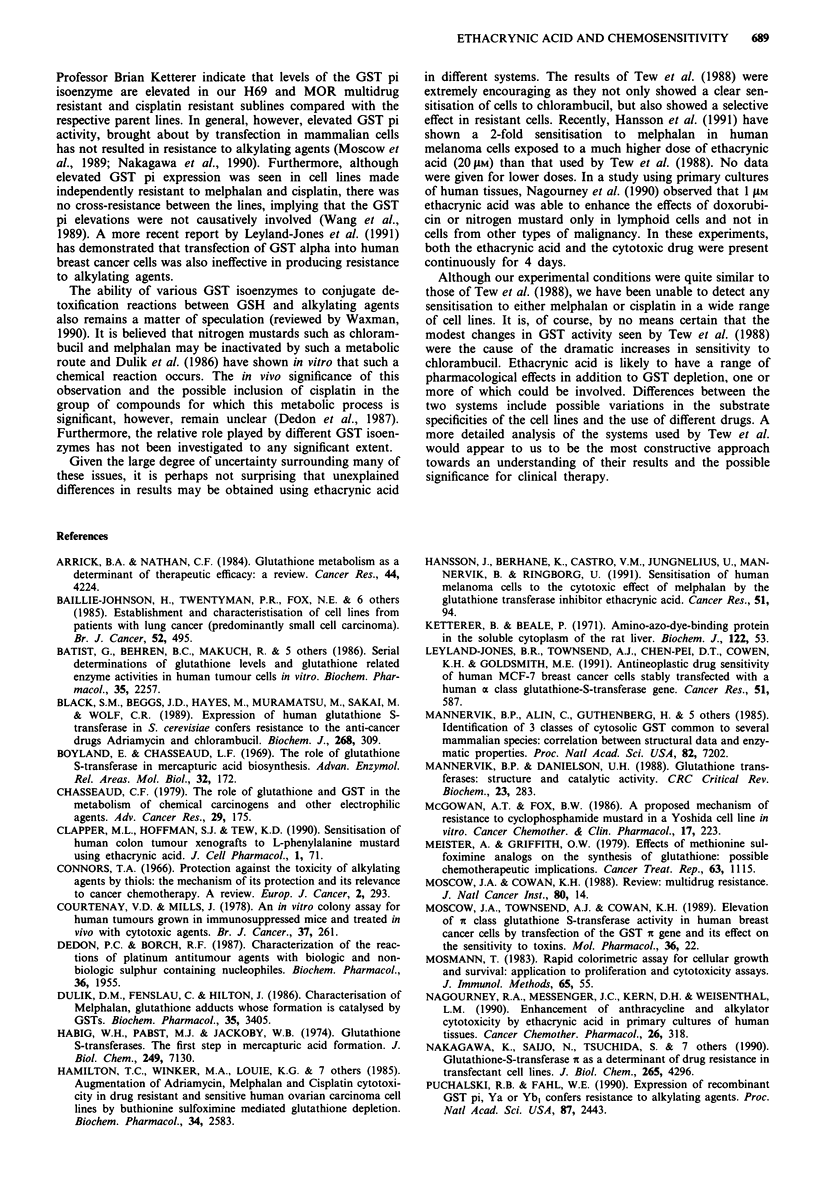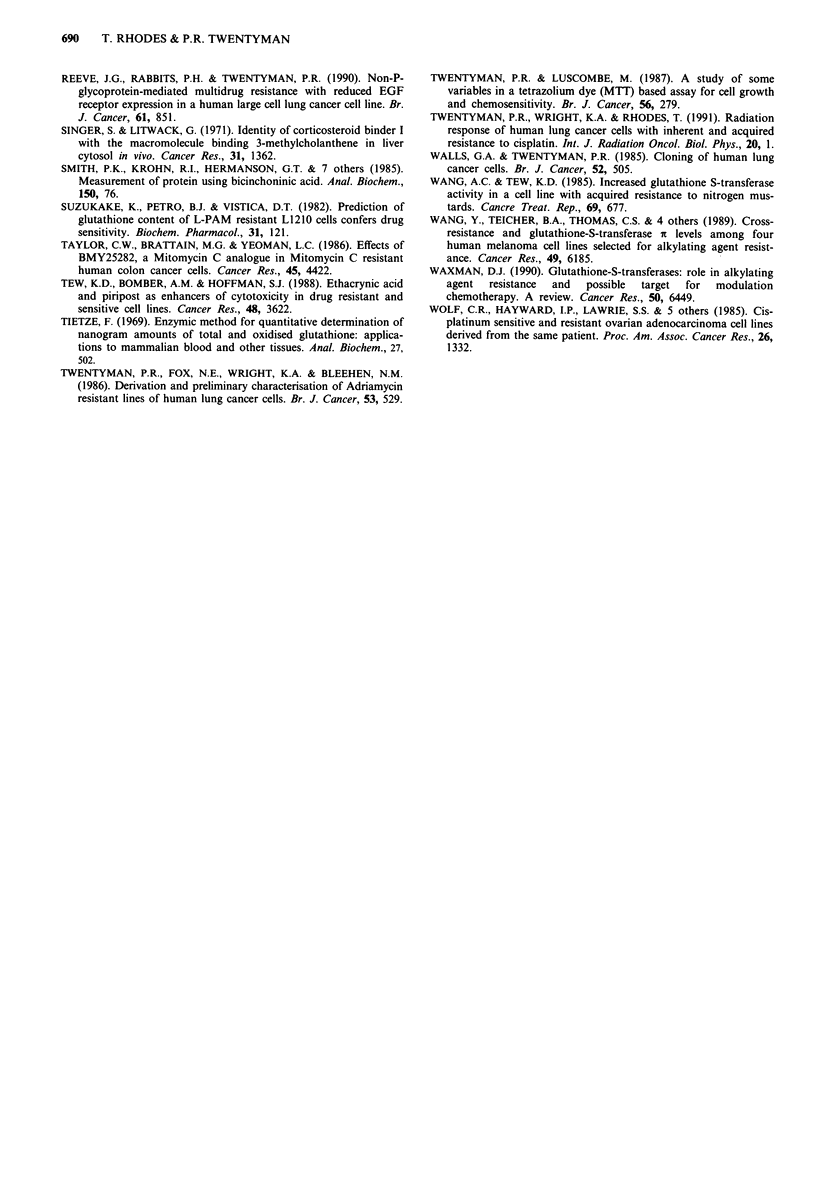# A study of ethacrynic acid as a potential modifier of melphalan and cisplatin sensitivity in human lung cancer parental and drug-resistant cell lines.

**DOI:** 10.1038/bjc.1992.145

**Published:** 1992-05

**Authors:** T. Rhodes, P. R. Twentyman

**Affiliations:** MRC Clinical Oncology and Radiotherapeutics Unit, Medical Research Council Centre, Cambridge, UK.

## Abstract

We have studied alterations in glutathione (GSH) levels and glutathione-S-transferase (GST) activity in a series of in vitro derived multidrug resistant and cisplatin resistant sublines of the human lung cancer lines NCI-H69 (small cell), COR-L23 (large cell) and MOR (adenocarcinoma). We have also investigated the effects of ethacrynic acid, a putative inhibitor of GSTs, on levels of GSH and GST activity and on cellular sensitivity to melphalan and to cisplatin. Neither GSH content nor GST activity were significantly greater in the resistant sublines compared with their respective parental lines. The only effects of treating with ethacrynic acid at doses of 1 microgram ml-1 and 3 micrograms ml-1 for 2 h were a reduction in GSH content in the cisplatin resistant subline H69/CPR at the 3 micrograms ml-1 dose, and an increase to over 140% of control at 1 microgram ml-1 and 3 micrograms ml-1 in the MOR parental line (MOR/P) and at 1 microgram ml-1 in the multidrug resistant subline MOR/R. Exposure of parental line COR-L23/P to 3 micrograms ml-1 and 6 micrograms ml-1 of ethacrynic acid for 24 h, however, increased the GSH content to over 300% and 500% of control respectively. Variable effects of ethacrynic acid on GST activity were seen in these cell lines. Doses of 1 microgram ml-1 and 3 micrograms ml-1 reduced activity to 59% and 48% of control respectively in multidrug resistant subline H69/LX4. On the other hand, activity was increased in the cisplatin resistant subline H69/CPR (to 146% and 218% of control) and in MOR/P (to 117% and 137% of control) by 1 microgram ml-1 and 3 micrograms ml-1 respectively of ethacrynic acid. Addition of ethacrynic acid (3 micrograms ml-1) to treatment of the cell lines with melphalan or with cisplatin did not alter the dose-response curves to these agents.


Br. J. Cancer (1992), 65, 684-690 © Macmillan Press Ltd., 1992

### A study of ethacrynic acid as a potential modifier of melphalan and cisplatin sensitivity in human lung cancer parental and drug-resistant cell lines

T. Rhodes & P.R. Twentyman

*MRC Clinical Oncology and Radiotherapeutics Unit, Medical Research Council Centre, Hills Road, Cambridge CB2 2QH, UK.*

**Summary** We have studied alterations in glutathione (GSH) levels and glutathione-S-transferase (GST) activity in a series of *in vitro* derived multidrug resistant and cisplatin resistant sublines of the human lung cancer lines NCI-H69 (small cell), COR-L23 (large cell) and MOR (adenocarcinoma). We have also investigated the effects of ethacrynic acid, a putative inhibitor of GSTs, on levels of GSH and GST activity and on cellular sensitivity to melphalan and to cisplatin.

Neither GSH content nor GST activity were significantly greater in the resistant sublines compared with their respective parental lines. The only effects of treating with ethacrynic acid at doses of $1\,\mu g\,ml^{-1}$ and $3\,\mu g\,ml^{-1}$ for 2 h were a reduction in GSH content in the cisplatin resistant subline H69/CPR at the $3\,\mu g\,ml^{-1}$ dose, and an increase to over 140% of control at $1\,\mu g\,ml^{-1}$ and $3\,\mu g\,ml^{-1}$ in the MOR parental line (MOR/P) and at $1\,\mu g\,ml^{-1}$ in the multidrug resistant subline MOR/R. Exposure of parental line COR-L23/P to $3\,\mu g\,ml^{-1}$ and $6\,\mu g\,ml^{-1}$ of ethacrynic acid for 24 h, however, increased the GSH content to over 300% and 500% of control respectively.

Variable effects of ethacrynic acid on GST activity were seen in these cell lines. Doses of $1\,\mu g\,ml^{-1}$ and $3\,\mu g\,ml^{-1}$ reduced activity to 59% and 48% of control respectively in multidrug resistant subline H69/LX4. On the other hand, activity was increased in the cisplatin resistant subline H69/CPR (to 146% and 218% of control) and in MOR/P (to 117% and 137% of control) by $1\,\mu g\,ml^{-1}$ and $3\,\mu g\,ml^{-1}$ respectively of ethacrynic acid.

Addition of ethacrynic acid ($3\,\mu g\,ml^{-1}$) to treatment of the cell lines with melphalan or with cisplatin did not alter the dose-response curves to these agents.

The intrinsic or acquired resistance of many tumours to chemotherapy is a major obstacle to the successful treatment of cancer. A number of mechanisms of cellular resistance have been shown to confer cross-resistance to groups of cytotoxic drugs (Moscow & Cowan, 1988). One such mechanism which leads to resistance to alkylating agents in cell lines involves elevated levels of glutathione (GSH) or changes in the activity of enzymes involved in GSH biochemistry (Meister & Griffith, 1979; Dulik *et al.*, 1986).

The tripeptide GSH is the principal intracellular non-protein thiol. The roles of GSH in cellular metabolism include protection from oxygen intermediates under aerobic metabolism and detoxification of metabolites (for review see Arrick & Nathan, 1984). It has been known for many years that thiols can protect cells from the toxicity of ionising radiation and alkylating agents (for review see Connors, 1966). Meister and Griffith (1979) suggested that reducing cellular GSH may be an approach to increasing the sensitivity of cells to alkylating agents. A number of workers have subsequently described cell lines in which drug resistance is associated with increased levels of cellular GSH and where reduction of GSH can restore drug sensitivity (Suzukake, 1982; Hamilton *et al.*, 1985; Batist *et al.*, 1986).

The detoxification of alkylating agents by GSH occurs via direct conjugation of the thiol group and the reactive alkylating group (Connors, 1966). This reaction is catalysed by a group of enzymes known as glutathione-S-transferase (GSTs) (Boyland & Chasseaud, 1969; Habig *et al.*, 1974). The GSTs also form covalent interactions with reactive metabolites of certain carcinogens resulting in detoxification (Singer & Litwack, 1971; Ketterer & Beale, 1971; Chasseaud, 1979). Soluble GSTs have been separated into three classes, alpha (basic), mu (neutral), and pi (acidic) on the basis of structural, immunological, and enzymatic properties (Mannervik *et al.*, 1985). Although studies have demonstrated some overlap in isoenzyme substrate specificity, the differential GST isoenzyme profiles of cell lines from different species and/or different tissues are likely to lead to variability of action against various substrates.

A number of cell lines made resistant *in vitro* to cytotoxic drugs have been shown to display increased GST activity. For example Wang and Tew (1985) found 2-5-fold elevated GST levels in a Walker 256 cell line made resistant to chlorambucil, compared to the sensitive line. Similar results have been found for cell lines resistant to cyclophosphamide (McGown & Fox, 1986), Adriamycin (Batist, 1986), and mitomycin C (Taylor, 1986). GST pi was found to be elevated in a series of drug resistant sublines of a human malignant melanoma cell line. This elevation did not however confer cross resistance to drugs used for the selection of other resistant sublines which also exhibited GST pi. (Wang *et al.*, 1989). Activity of GSTs have also been found to be increased in human ovarian tumour tissues which are resistant to chemotherapy (Wolf *et al.*, 1985).

The transfection of genes encoding for human pi and alpha class GSTs into yeast cells has been shown to confer resistance to chlorambucil (Black *et al.*, 1989). Human GST pi genes have also been transfected into mammalian cells and found to confer resistance to known substrates of this class, such as ethacrynic acid, but not to anti-tumour agents such as cisplatin and melphalan (Moscow *et al.*, 1989). Puchalski and Fahl (1990) transfected rat alpha and mu class GST genes, as well as human pi class. The rat mu class conferred the greatest (albeit modest) resistance to cisplatin (1.5-fold).

Inhibitors of GST activity have been a focus of study as a possible means of reducing resistance (Mannervik & Danielson, 1988). One compound of particular interest has been ethacrynic acid, a clinically used diuretic agent (Tew *et al.*, 1988). Ethacrynic acid has been shown to increase cell kill by chlorambucil or melphalan in Walker 256 and HT29 *in vitro* (Tew *et al.*, 1988) and human tumours xenografted into nude mice (Clapper *et al.*, 1990).

In this paper we describe experiments designed to investigate the possible role of GSH and/or GST activity in inherent or acquired resistance of human lung cancer cell lines. We

Correspondence: T. Rhodes.
Received 7 October 1991; and in revised form 20 December 1991.

then examine the effects of ethacrynic acid upon GSH, GST and sensitivity to melphalan or cisplatin.

### Materials and methods

### *Cell lines and culture conditions*

The NCI-H69 human small cell lung cancer line (hereafter referred to as H69/P) originally supplied by Drs D. Carney and A. Gazdar (NCI/Navy Medical Oncology Branch, Bethesda, MD, USA) was grown as floating aggregates in RPMI 1640 medium (Gibco Biocult, Paisley, UK) supplemented with 10% foetal calf serum (Seralab, Crawley Down, UK), penicillin and streptomycin (at concentrations of 100 IU $ml^{-1}$ and 100 $\mu g\ ml^{-1}$ respectively). Stock cultures were maintained in 75 $cm^2$ tissue-culture flasks (Falcon Plastics, Cambridge, UK) at 37°C in an atmosphere of 92% air and 8% $CO_2$. The large cell lung cancer line, COR-L23 (L23/P) (Baillie-Johnson *et al.*, 1985) and the adenocarcinoma line MOR (MOR/P) (supplied by Dr M. Ellison, Ludwig Inst. Sutton Branch) were grown as monolayers on plastic and were maintained under the same medium and culture conditions as H69/P. Subculture of H69/P and resistant sublines was achieved by mechanical dissagregation and transfer of small groups of cells to new flasks, whereas for L23/P, MOR/P and resistant sublines, the use of 0.4% trypsin plus 0.02% versene was required.

### *Drug resistant sublines*

The drug resistant sublines of the H69/P, L23/P and MOR/P lines were maintained under the same culture conditions as the parent lines, except for the addition of various concentrations of drugs to the growth medium. Drug resistance was developed in each case by the addition of a stepwise increase in drug concentration to the growth medium of the parental line as previously described. The H69/LX4 subline, an MDR cell line which exhibits an 85-fold resistance to Adriamycin (Twentyman *et al.*, 1986) and hyperexpresses P-glycoprotein (Reeve *et al.*, 1989) was maintained at 0.4 $\mu g\ ml^{-1}$ of Adriamycin. The H69/CPR subline, which exhibits a 5-fold resistance to cisplatin and is cross resistant to melphalan (Twentyman *et al.*, 1991) was maintained in cisplatin at a concentration of 0.4 $\mu g\ ml^{-1}$. L23/R, a subline which expresses an MDR phenotype without hyperexpression of P-glycoprotein (Twentyman *et al.*, 1986; Reeve *et al.*, 1990) and which is 20-fold resistant to Adriamycin, was grown in 0.2 $\mu g\ ml^{-1}$ Adriamycin and L23/CPR, which exhibits a 3-fold resistance to cisplatin and is cross resistant to melphalan (Twentyman *et al.*, 1991) was grown in 0.05 $\mu g\ ml^{-1}$ cisplatin. MOR/R which also expresses an MDR phenotype without P-glycoprotein hyperexpression (Twentyman *et al.*, 1986 and unpublished) exhibits a 10-fold resistance to Adriamycin and was maintained in 0.2 $\mu g\ ml^{-1}$ of Adriamycin and MOR/CPR which exhibits a 4-fold resistance to cisplatin and is cross resistant to melphalan (Twentyman *et al.*, 1991) was maintained in 1 $\mu g\ ml^{-1}$ cisplatin. All resistant cells were grown in drug-free medium for at least 3 days before use in experiments.

### *Drugs*

Ethacrynic acid (Sigma, Poole, UK) was dissolved in absolute ethanol. Melphalan (Wellcome Foundation Ltd, London, UK) was dissolved in acidified ethanol. These two agents were freshly prepared immediately before use and the final concentration of ethanol in the medium did not exceed 0.2%. Cis-diamminedichloroplatinum (II) (cisplatin) (Lederle, Gosport, UK) was dissolved in distilled water and aliquots were stored at − 20°C. Drug was added to cells immediately following thawing. Appropriate solvent controls were used in all experiments.

### *Biochemical assays*

Cells of the L23 and MOR parent and resistant lines, were subcultured from stock flasks and inoculated into 25 $cm^2$ flasks 4 days before experiments. Cells were in exponential growth at the time of assay and a medium change was carried out 24 h before assay. Ethacrynic acid was added to produce a range of final concentrations and after 2 h (except where a time course was being studied) the monolayer was rinsed three times with PBS and cells then disaggregated and counted as above.

Cultures of H69/P and resistant sublines in exponential phase of growth, containing aggregates of various sizes, were pipetted well in order to break up the aggregates, and medium changed 24 h before assay. This resulted in cultures containing single cells and small groups of cells at the time of assay. Immediately before drug treatment, a sample of the culture was removed, disaggregated into a single cell suspension using 15 min incubation with 0.4% trypsin and 0.02% versene and a count carried out. On the basis of the count the bulk culture was diluted and aliquots were transferred into 10 ml of plastic centrifuge tubes for experiments. The cells were treated for 2 h with various concentrations of ethacrynic acid and then rinsed three times with PBS by centrifugation.

*GSH assay (oxidised and reduced)* Cells were transferred into plastic centrifuge tubes, rinsed three times with ice-cold PBS and lysed using 100% TCA. They were then centrifuged at 4°C (11,000 *g* for 10 min) and the supernatant was then removed and used for total GSH analysis by the method of Tietze (1969). Protein was removed by five cycles of diethyl ether extraction followed by the complete evaporation of diethyl ether from the sample. The reaction mixture consisted of 5,5′ dithio-bis-(2-nitrobenzoic acid) (DTNB), 0.15 $\mu$mole in 100 $\mu$l, nicotinamide adenine dinucleotide phosphate (reduced form) (NADPH), 0.2 $\mu$mole in 100 $\mu$l, glutathione reductase (1 unit in 50 $\mu$l), the sample, blank or standard in a 50 $\mu$l volume and all made up to 1 ml with phosphate EDTA buffer pH 7.5. The reaction took place at 30°C and the rate of colour development was read at 412 nm over a period of 6 min. All reagents were obtained from Sigma (Poole, UK).

*GST assay* Assay for GST was carried out by the method of Habig *et al.* (1974) using 1-chloro-2,4-dinitrobenzene as a substrate. Cells were sonicated, centrifuged at 18,000 *g* for 20 min, and the supernatant used for the kinetic studies. Reaction mixture consisted of 940 $\mu$l of 1 mM CDNB (made up by dissolving in 4 ml ethanol and adding drop by drop to 196 ml 0.1 M phospate buffer pH 6.5), 50 $\mu$l of 20 mM GSH dissolved in distilled water, and 10 $\mu$l sample. The colour development was read at 340 nm over a 2 min period at 25°C. All reagents were obtained from Sigma (Poole, UK).

*Protein assay* Total cytosolic protein determinations were carried out, following sonication of cells, by the Bicinchoninic acid assay (BCA), using a kit from Pierce (Luton, UK) (Smith *et al.*, 1985).

### *Chemosensitivity assays*

Cellular response to treatment with ethacrynic acid or with cytotoxic drugs was determined either by the MTT colorimetric assay or by clonogenic assay.

*MTT assay* The assay used was based on that described by Mosmann (1983), and modified in this laboratory (Twentyman & Luscombe, 1987). Cells were plated into 96-well microtitre plates (Falcon Plastics, Cambridge, UK) at $4 \times 10^3$ (H69/P), $5 \times 10^3$ (H69/LX4), $5 \times 10^3$ (H69/CPR), $1 \times 10^3$ (L23/P), $2 \times 10^3$ (L23/R), $4 \times 10^3$ (L23/CPR), $4 \times 10^3$ (MOR/P), $6 \times 10^3$ (MOR/R), $5 \times 10^3$ (MOR/CPR) per well, in 200 $\mu$l medium. After a period of approximately 2 h incubation (8% $CO_2$, 92% air, 37°C) 20 $\mu$l of the appropriate concentration of solvent or ethacrynic acid was added to the plates. Groups of plates were then incubated either for a period of 6 days ('continuous exposure') in a gassing incubator (8% $CO_2$, 92% air, 37°C) or for 24 h followed by medium change, including two rinsing cycles, and a further 5 days incubation in drug free conditions.

At the end of the incubation period, 20 µl of a 5 mg ml$^{-1}$ solution of 3-(4, 5-Dimethylthiazol-2-yl)-2,5-diphenyltetrazolium bromide (MTT) (Sigma, Poole, UK) in PBS was added to each well and the plates returned to the incubator for a further 5 h. After this, plates containing cells which grow as floating aggregates were centrifuged for 5 min at 400 *g* in order to pack the floating aggregates to the bottom of the wells, whilst plates from adherent lines were not centrifuged. The bulk of the medium was removed from each well using a Pasteur pipette connected to a vacuum line, leaving 10–20 µl medium per well. To each well was then added 200 µl DMSO (BDH, Poole, UK) and the plates were agitated on a plate shaker for 10 min. Optical densities were then read at 540 nm and a reference wavelength of 690 nm on a Titertek Multiskan MCC ELISA plate reader (Flow Laboratories, Rickmansworth, UK). Results were expressed as a fraction of control absorbances.

*Clonogenic assay* Cells were treated with drugs either in monolayer (L23/P, L23/R, L23/CPR, MOR/P, MOR/R and MOR/CPR) or as aggregates in suspension (H69/P, H69/LX4, H69/CPR) as described in 'biochemical assays' (above). The soft agar clonogenic assay used was that of Courtenay and Mills, (1978) with some modification (Walls & Twentyman, 1985). Briefly, known numbers of cells were plated into tubes in Ham's F12 medium without 0.3% agar and August rat red blood cells. Tubes were gassed with 5% $CO_2$, 5% $O_2$ and 90% $N_2$ and sealed. Racks of tubes were placed into plastic boxes which were in turn gassed and sealed. Tubes were incubated at 37°C and fed weekly with Ham's F12 medium, for 2–3 weeks depending on the cell line. Excess medium was removed from the tubes and agar plugs were fixed with 3% formaldehyde in PBS. Colonies containing $>50$ cells were counted by squashing each plus on a petri dish and examining it with an inverted microscope.

### Results

### *GSH content*

GSH content was measured by the Tietze assay in H69/P, L23/P, MOR/P and the drug resistant sublines, growing in exponential phase (Table I). None of the resistant cell lines showed significantly elevated levels of GSH. L23/R (multidrug resistant) contained half the amount of GSH present in the line from which it was derived (L23/P). GSH content was also reduced in the multidrug and cisplatin resistant sublines of the MOR line, but these differences were not statistically significant. There were large differences in GSH content between the various parent lung cancer lines with MOR/P having the highest content (approximately twice that of L23/P and ten times higher than H69/P) on a per cell basis.

**Table I** GSH levels and GST activity in human lung cancer cell lines

| Cell line | GSH [ng $(10^4$ cells$)^{-1}$] | GST activity [nmole CDNB conjugated (mg protein)$^{-1}$) (min)$^{-1}$] |
|---|---|---|
| H69/P | 14.6 (3.7) | 63.7 (14.8) |
| H69/LX4 | 14.0 (4.4) | 86.3 (30.8) |
| H69/CPR | 17.3 (7.9) | 108.6 (42.9) |
| L23/P | 74.2 (30.2) | 41.6 (16.0) |
| L23/R | 31.7 (13.2) | 9.2 (4.6) |
| L23/CPR | 50.7 (14.3) | 16.3 (17.1) |
| MOR/P | 124.0 (52.7) | 359.0 (123.4) |
| MOR/R | 97.0 (59.7) | 396.0 (175.3) |
| MOR/CPR | 98.8 (24.4) | 385.5 (157.5) |

Values are the means of typically five separate determinations, numbers in brackets are standard deviations.

### *GST activity*

Measurements of GST activity in cell lines in exponential growth phase, by conjugation of CDNB, revealed increased levels in the multidrug resistant subline of H69/P and further increases in the cisplatin resistant subline, these increases were however not statistically significant. Conversely, levels in the multidrug resistant subline L23/R were significantly reduced. There was also a reduction in the cisplatin resistant line L23/CPR, but this was not statistically significant. The MOR parent cell line showed 5.6-fold greater activity than H69/P and 8.6-fold greater activity than L23/P but there were no significant differences between the parent and resistant lines MOR (Table I).

### *Effect of ethacrynic acid alone*

Cells were exposed to various doses of ethacrynic acid either throughout the 6 day assay or for 24 h followed by a further 5 days incubation after extensive rinsing. MTT data in Figure 1 show that the sensitivities of the parental and drug resistant cell lines to ethacrynic acid were rather similar, except that both the multidrug resistant (H69/LX4) and the cisplatin resistant (H69/CPR) sublines of H69/P were more sensitive than the parent line to the 24 h exposure. MOR lines were more generally resistant than the others particularly in continuous exposure experiments.

In experiments in which cells were exposed to ethacrynic

**Figure 1** The effect of ethacrynic acid on parent (○), multidrug resistant (●), and cisplatin resistant (□) human lung cancer cell lines, NCI-H69 (**a**,**b**), COR-L23 (**c**,**d**), and MOR (**e**,**f**). Assessed in a 6 day MTT assay following 24 h drug exposure (**a**,**c**,**e**) or continuous drug exposure (**b**,**d**,**f**). Each point is based on four replicate wells.

acid (doses up to 10 μg ml $^{-1}$) for 2 h followed by clonogenic assay, reduction in cell survival to less than 50% of control was not seen in any of the cell lines (data not shown).

Table II shows GSH levels or GST activity after treatment of cells with ethacrynic acid at 1 or 3 μg ml $^{-1}$ for 2 h. Glutathione levels were increased as a result of this treatment in all cell lines except H69/CPR and MOR/CPR. The effect of prolonging ethacrynic acid exposure on L23/P was that levels of GSH were increased. The results in Figure 2 show that doses of 3 μg ml $^{-1}$ and 6 μg ml $^{-1}$ increase levels to three and five times the control respectively after 24 h exposure. These increased GSH levels were detected only after at least 8 h exposure to ethacrynic acid.

The effect of 2 h ethacrynic acid treatment on GST activity was very varied. Activity was decreased to less than 80% of control in H69/P and H69/LX4 at 1 μg ml $^{-1}$ and also in H69/LX4 at 3 μg ml $^{-1}$. Conversely, activity was increased to greater than 120% of control in H69/CPR at 1 μg ml $^{-1}$ and in H69/CPR, L23/CPR and MOR/P at 3 μg ml $^{-1}$.

### *Effects of ethacrynic acid in combination with cisplatin and melphalan*

The results of typical combination experiments in which cells were treated for 2 h with 3 μg ml $^{-1}$ ethacrynic acid, with either cisplatin or melphalan also present during the second hour are summarised in Figures 3 to 5. It may be be seen that no clear enhancement of cytotoxic drug effects were seen for any of the combinations. Combination of ethacrynic acid with cisplatin and melphalan was studied in all cell lines on at least two independent occasions, a total of 22 experiments. No clear potentiation of melphalan or cisplatin was seen in any of these experiments.

**Figure 3** The effect of treating L23 parent (**a**), multidrug resistant (**b**) and cisplatin resistant (**c**) cell lines for 1 h either alone (●) or in combination with ethacrynic acid (3 μg ml$^{-1}$) for 1 h before and during melphalan exposure (O). Error bars show standard errors based on Poisson errors in total colony count in three replicate tubes.

**Table II** GSH levels and GST activity in human lung cancer cell lines following treatment with ethacrynic acid for 2 h at 1 or 3 μg ml$^{-1}$

| Cell line | *GSH/cell (% control)* 1 μg ml$^{-1}$ | 3 μg ml$^{-1}$ | *GST activity (% control)* 1 μg ml$^{-1}$ | 3 μg ml$^{-1}$ |
|---|---|---|---|---|
| 69/P | 119 | 102 | 74 | 119 |
| H69/LX4 | 109 | 108 | 59 | 48 |
| H69/CPR | 111 | 65 | 146 | 218 |
| L23/P | 100 | 110 | 93 | 119 |
| L23/R | 131 | 125 | 110 | 111 |
| L23/CPR | 122 | 120 | 93 | 135 |
| MOR/P | 143 | 144 | 117 | 137 |
| MOR/R | 152 | 110 | 110 | 111 |
| MOR/CPR | 107 | 94 | 88 | 81 |

Values are the means of at least two separate determinations.

**Figure 2** The effect with time of exposure to ethacrynic acid at 3 μg ml$^{-1}$ (O) or 6 μg ml$^{-1}$ (●) on the GSH content of the L23/P cell line.

### Discussion

We commenced this study in the hope that the use of ethacrynic acid would prove to be an effective means of overcoming acquired resistance to melphalan and/or cisplatin in human lung cancer cell lines and eventually in the clinic. The data presented in this paper do not, however, support the hypothesis that ethacrynic acid is able to increase the efficacy of alkylating agent cytotoxicity and are therefore in marked contrast to those reported previously by Tew *et al.* (1988) for chlorambucil.

In the earlier study, it was found that (a) 2 h treatment with 1 μg ml $^{-1}$ (3μM) ethacrynic acid could produce a modest depletion in GST activity and GSH levels in a range of cell lines and (b) the response to chlorambucil, present for the second hour of such a pretreatment could be dramatically enhanced by this dose of ethacrynic acid. It was particularly noticeable that the enhancement in a resistant Walker tumour cell line was much greater than in a sensitive line, although the depletion of GST activity (in percentage terms) was similar and the activity (measured using CDNB as substrate) was 3.5-fold higher in the resistant line. In a subsequent paper, the same authors reported enhancement of melphalan response by ethacrynic acid in human colon car-

**Figure 4** The effect of treating H69 parent (**a**,**c**) and cisplatin resistant (**b**,**d**) cell lines for 1 h with melphalan (**a**,**b**) alone (●) or in combinatin with ethacrynic acid (3 $\mu g\ ml^{-1}$) for 1 h before and during cytotoxic drug exposure (○). Error bars show standard error based on Poisson errors in total colony count in three replicate tubes.

**Figure 5** The effects of treating MOR parent, multidrug resistant and cisplatin resistant cell lines for 1 h with either 5 or 20 $\mu g\ ml^{-1}$ of cisplatin alone (■) or in combination with ethacrynic acid (3 $\mu g\ ml^{-1}$) for 1 h before and during cisplatin exposure (▨). Error bars show standard errors based on Poisson errors in total colony count in three replicate tubes.

cinoma cells growing as xenografts in nude mice (Clapper *et al.*, 1990).

Interpretaton of the various data sets are complicated by a number of factors. Firstly, there are potential effects of ethacrynic acid on both GSH levels and GST activity. Each of these factors is known to be independently involved in determining alkylating agent sensitivity. Tew *et al.* (1988) reported that in HT29 colon carcinoma cells chronically exposed to low dose ethacrynic acid, an elevation of GST activity was seen, but that there was no increase in GSH. Conversely, we found that a prolonged exposure to ethacrynic acid leads to a clear increase in GSH levels in human lung cancer cells after some hours. In order to minimise these complicating effects we decided, as did Tew *et al.* (1988), to concentrate on short time exposures in our combination experiments.

We found the effects of 2 h exposure to ethacrynic acid to be somewhat variable in terms of changes in GSH and GST activity, despite our efforts to ensure that our cells were in similar growth states in each experiment. Our data indicate the effects to be cell line dependent. Whilst, for instance, we saw a decrease in GST activity in the subline H69/LX4, we saw an increase in a different subline, H69/CPR. Because of the variability however, these differences do not reach statistical significance.

The baseline levels of GSH and GST activity in the various sublines need to be considered. Whereas none of the drug-resistant sublines exhibit clear changes in GSH content from their parent lines, several sublines show marked changes in GST activity. The cisplatin (and melphalan) resistant subline H69/CPR shows a 60% increase in GST activity compared with its parent line, whilst L23/R (multidrug resistant) and L23/CPR (cisplatin and melphalan resistant) show 4.5-fold and 2.5-fold reductions respectively compared to the parent line L23/P. We are uncertain as to whether or not these changes are responsible to any extent for the changed drug sensitivity profiles of these sublines. Preliminary unpublished data obtained in collaboration with Dr Jonathan Harris and

Professor Brian Ketterer indicate that levels of the GST pi isoenzyme are elevated in our H69 and MOR multidrug resistant and cisplatin resistant sublines compared with the respective parent lines. In general, however, elevated GST pi activity, brought about by transfection in mammalian cells has not resulted in resistance to alkylating agents (Moscow et al., 1989; Nakagawa et al., 1990). Furthermore, although elevated GST pi expression was seen in cell lines made independently resistant to melphalan and cisplatin, there was no cross-resistance between the lines, implying that the GST pi elevations were not causatively involved (Wang et al., 1989). A more recent report by Leyland-Jones et al. (1991) has demonstrated that transfection of GST alpha into human breast cancer cells was also ineffective in producing resistance to alkylating agents.

The ability of various GST isoenzymes to conjugate detoxification reactions between GSH and alkylating agents also remains a matter of speculation (reviewed by Waxman, 1990). It is believed that nitrogen mustards such as chlorambucil and melphalan may be inactivated by such a metabolic route and Dulik et al. (1986) have shown *in vitro* that such a chemical reaction occurs. The *in vivo* significance of this observation and the possible inclusion of cisplatin in the group of compounds for which this metabolic process is significant, however, remain unclear (Dedon et al., 1987). Furthermore, the relative role played by different GST isoenzymes has not been investigated to any significant extent.

Given the large degree of uncertainty surrounding many of these issues, it is perhaps not surprising that unexplained differences in results may be obtained using ethacrynic acid in different systems. The results of Tew et al. (1988) were extremely encouraging as they not only showed a clear sensitisation of cells to chlorambucil, but also showed a selective effect in resistant cells. Recently, Hansson et al. (1991) have shown a 2-fold sensitisation to melphalan in human melanoma cells exposed to a much higher dose of ethacrynic acid (20 $\mu$M) than that used by Tew et al. (1988). No data were given for lower doses. In a study using primary cultures of human tissues, Nagourney et al. (1990) observed that 1 $\mu$M ethacrynic acid was able to enhance the effects of doxorubicin or nitrogen mustard only in lymphoid cells and not in cells from other types of malignancy. In these experiments, both the ethacrynic acid and the cytotoxic drug were present continuously for 4 days.

Although our experimental conditions were quite similar to those of Tew et al. (1988), we have been unable to detect any sensitisation to either melphalan or cisplatin in a wide range of cell lines. It is, of course, by no means certain that the modest changes in GST activity seen by Tew et al. (1988) were the cause of the dramatic increases in sensitivity to chlorambucil. Ethacrynic acid is likely to have a range of pharmacological effects in addition to GST depletion, one or more of which could be involved. Differences between the two systems include possible variations in the substrate specificities of the cell lines and the use of different drugs. A more detailed analysis of the systems used by Tew et al. would appear to us to be the most constructive approach towards an understanding of their results and the possible significance for clinical therapy.

### References

ARRICK, B.A. & NATHAN, C.F. (1984). Glutathione metabolism as a determinant of therapeutic efficacy: a review. *Cancer Res.*, **44**, 4224.

BAILLIE-JOHNSON, H., TWENTYMAN, P.R., FOX, N.E. & 6 others (1985). Establishment and characteristisation of cell lines from patients with lung cancer (predominantly small cell carcinoma). *Br. J. Cancer*, **52**, 495.

BATIST, G., BEHREN, B.C., MAKUCH, R. & 5 others (1986). Serial determinations of glutathione levels and glutathione related enzyme activities in human tumour cells *in vitro*. *Biochem. Pharmacol.*, **35**, 2257.

BLACK, S.M., BEGGS, J.D., HAYES, M., MURAMATSU, M., SAKAI, M. & WOLF, C.R. (1989). Expression of human glutathione S-transferase in *S. cerevisiae* confers resistance to the anti-cancer drugs Adriamycin and chlorambucil. *Biochem. J.*, **268**, 309.

BOYLAND, E. & CHASSEAUD, L.F. (1969). The role of glutathione S-transferase in mercapturic acid biosynthesis. *Advan. Enzymol. Rel. Areas. Mol. Biol.*, **32**, 172.

CHASSEAUD, C.F. (1979). The role of glutathione and GST in the metabolism of chemical carcinogens and other electrophilic agents. *Adv. Cancer Res.*, **29**, 175.

CLAPPER, M.L., HOFFMAN, S.J. & TEW, K.D. (1990). Sensitisation of human colon tumour xenografts to L-phenylalanine mustard using ethacrynic acid. *J. Cell Pharmacol.*, **1**, 71.

CONNORS, T.A. (1966). Protection against the toxicity of alkylating agents by thiols: the mechanism of its protection and its relevance to cancer chemotherapy. A review. *Europ. J. Cancer*, **2**, 293.

COURTENAY, V.D. & MILLS, J. (1978). An *in vitro* colony assay for human tumours grown in immunosuppressed mice and treated *in vivo* with cytotoxic agents. *Br. J. Cancer.*, **37**, 261.

DEDON, P.C. & BORCH, R.F. (1987). Characterization of the reactions of platinum antitumour agents with biologic and non-biologic sulphur containing nucleophiles. *Biochem. Pharmacol.*, **36**, 1955.

DULIK, D.M., FENSLAU, C. & HILTON, J. (1986). Characterisation of Melphalan, glutathione adducts whose formation is catalysed by GSTs. *Biochem. Pharmacol.*, **35**, 3405.

HABIG, W.H., PABST, M.J. & JACKOBY, W.B. (1974). Glutathione S-transferases. The first step in mercapturic acid formation. *J. Biol. Chem.*, **249**, 7130.

HAMILTON, T.C., WINKER, M.A., LOUIE, K.G. & 7 others (1985). Augmentation of Adriamycin, Melphalan and Cisplatin cytotoxicity in drug resistant and sensitive human ovarian carcinoma cell lines by buthionine sulfoximine mediated glutathione depletion. *Biochem. Pharmacol.*, **34**, 2583.

HANSSON, J., BERHANE, K., CASTRO, V.M., JUNGNELIUS, U., MANNERVIK, B. & RINGBORG, U. (1991). Sensitisation of human melanoma cells to the cytotoxic effect of melphalan by the glutathione transferase inhibitor ethacrynic acid. *Cancer Res.*, **51**, 94.

KETTERER, B. & BEALE, P. (1971). Amino-azo-dye-binding protein in the soluble cytoplasm of the rat liver. *Biochem. J.*, **122**, 53.

LEYLAND-JONES, B.R., TOWNSEND, A.J., CHEN-PEI, D.T., COWEN, K.H. & GOLDSMITH, M.E. (1991). Antineoplastic drug sensitivity of human MCF-7 breast cancer cells stably transfected with a human $\alpha$ class glutathione-S-transferase gene. *Cancer Res.*, **51**, 587.

MANNERVIK, B.P., ALIN, C., GUTHENBERG, H. & 5 others (1985). Identification of 3 classes of cytosolic GST common to several mammalian species: correlation between structural data and enzymatic properties. *Proc. Natl Acad. Sci. USA*, **82**, 7202.

MANNERVIK, B.P. & DANIELSON, U.H. (1988). Glutathione transferases: structure and catalytic activity. *CRC Critical Rev. Biochem.*, **23**, 283.

MCGOWAN, A.T. & FOX, B.W. (1986). A proposed mechanism of resistance to cyclophosphamide mustard in a Yoshida cell line *in vitro*. *Cancer Chemother. & Clin. Pharmacol.*, **17**, 223.

MEISTER, A. & GRIFFITH, O.W. (1979). Effects of methionine sulfoximine analogs on the synthesis of glutathione: possible chemotherapeutic implications. *Cancer Treat. Rep.*, **63**, 1115.

MOSCOW, J.A. & COWAN, K.H. (1988). Review: multidrug resistance. *J. Natl Cancer Inst.*, **80**, 14.

MOSCOW, J.A., TOWNSEND, A.J. & COWAN, K.H. (1989). Elevation of $\pi$ class glutathione S-transferase activity in human breast cancer cells by transfection of the GST $\pi$ gene and its effect on the sensitivity to toxins. *Mol. Pharmacol.*, **36**, 22.

MOSMANN, T. (1983). Rapid colorimetric assay for cellular growth and survival: application to proliferation and cytotoxicity assays. *J. Immunol. Methods*, **65**, 55.

NAGOURNEY, R.A., MESSENGER, J.C., KERN, D.H. & WEISENTHAL, L.M. (1990). Enhancement of anthracycline and alkylator cytotoxicity by ethacrynic acid in primary cultures of human tissues. *Cancer Chemother. Pharmacol.*, **26**, 318.

NAKAGAWA, K., SAIJO, N., TSUCHIDA, S. & 7 others (1990). Glutathione-S-transferase $\pi$ as a determinant of drug resistance in transfectant cell lines. *J. Biol. Chem.*, **265**, 4296.

PUCHALSKI, R.B. & FAHL, W.E. (1990). Expression of recombinant GST pi, Ya or $Yb_1$ confers resistance to alkylating agents. *Proc. Natl Acad. Sci. USA*, **87**, 2443.

REEVE, J.G., RABBITS, P.H. & TWENTYMAN, P.R. (1990). Non-P-glycoprotein-mediated multidrug resistance with reduced EGF receptor expression in a human large cell lung cancer cell line. *Br. J. Cancer*, **61**, 851.

SINGER, S. & LITWACK, G. (1971). Identity of corticosteroid binder I with the macromolecule binding 3-methylcholanthene in liver cytosol *in vivo*. *Cancer Res.*, **31**, 1362.

SMITH, P.K., KROHN, R.I., HERMANSON, G.T. & 7 others (1985). Measurement of protein using bicinchoninic acid. *Anal. Biochem.*, **150**, 76.

SUZUKAKE, K., PETRO, B.J. & VISTICA, D.T. (1982). Prediction of glutathione content of L-PAM resistant L1210 cells confers drug sensitivity. *Biochem. Pharmacol.*, **31**, 121.

TAYLOR, C.W., BRATTAIN, M.G. & YEOMAN, L.C. (1986). Effects of BMY25282, a Mitomycin C analogue in Mitomycin C resistant human colon cancer cells. *Cancer Res.*, **45**, 4422.

TEW, K.D., BOMBER, A.M. & HOFFMAN, S.J. (1988). Ethacrynic acid and piripost as enhancers of cytotoxicity in drug resistant and sensitive cell lines. *Cancer Res.*, **48**, 3622.

TIETZE, F. (1969). Enzymic method for quantitative determination of nanogram amounts of total and oxidised glutathione: applications to mammalian blood and other tissues. *Anal. Biochem.*, 27, 502.

TWENTYMAN, P.R., FOX, N.E., WRIGHT, K.A. & BLEEHEN, N.M. (1986). Derivation and preliminary characterisation of Adriamycin resistant lines of human lung cancer cells. *Br. J. Cancer*, **53**, 529.

TWENTYMAN, P.R. & LUSCOMBE, M. (1987). A study of some variables in a tetrazolium dye (MTT) based assay for cell growth and chemosensitivity. *Br. J. Cancer*, **56**, 279.

TWENTYMAN, P.R., WRIGHT, K.A. & RHODES, T. (1991). Radiation response of human lung cancer cells with inherent and acquired resistance to cisplatin. *Int. J. Radiation Oncol. Biol. Phys.*, **20**, 1.

WALLS, G.A. & TWENTYMAN, P.R. (1985). Cloning of human lung cancer cells. *Br. J. Cancer*, **52**, 505.

WANG, A.C. & TEW, K.D. (1985). Increased glutathione S-transferase activity in a cell line with acquired resistance to nitrogen mustards. *Cancre Treat. Rep.*, **69**, 677.

WANG, Y., TEICHER, B.A., THOMAS, C.S. & 4 others (1989). Cross-resistance and glutathione-S-transferase π levels among four human melanoma cell lines selected for alkylating agent resistance. *Cancer Res.*, **49**, 6185.

WAXMAN, D.J. (1990). Glutathione-S-transferases: role in alkylating agent resistance and possible target for modulation chemotherapy. A review. *Cancer Res.*, **50**, 6449.

WOLF, C.R., HAYWARD, I.P., LAWRIE, S.S. & 5 others (1985). Cis-platinum sensitive and resistant ovarian adenocarcinoma cell lines derived from the same patient. *Proc. Am. Assoc. Cancer Res.*, **26**, 1332.